# Delineating immune variation between adult and children COVID-19 cases and associations with disease severity

**DOI:** 10.1038/s41598-024-55148-9

**Published:** 2024-03-01

**Authors:** Alper Cevirgel, Martijn Vos, Anne Floor Holtrop, Lisa Beckers, Daphne F. M. Reukers, Adam Meijer, Nynke Rots, Josine van Beek, Debbie van Baarle, Jelle de Wit

**Affiliations:** 1 Center for Infectious Disease Control, National Institute for Public Health and the Environment, Bilthoven, The Netherlands; 2 Department of Medical Microbiology and Infection Prevention, Virology and Immunology Research Group, University Medical Center Groningen, Groningen, The Netherlands

**Keywords:** Paediatric research, Adaptive immunity, Infectious diseases, Innate immune cells

## Abstract

The SARS-CoV-2 pandemic has emphasized the need to explore how variations in the immune system relate to the severity of the disease. This study aimed to explore inter-individual variation in response to SARS-CoV-2 infection by comparing T cell, B cell, and innate cell immune subsets among primary infected children and adults (i.e., those who had never experienced SARS-CoV-2 infection nor received vaccination previously), with varying disease severity after infection. We also examined immune subset kinetics in convalescent individuals compared to those with persistent infection to identify possible markers of immune dysfunction. Distinct immune subset differences were observed between infected adults and children, as well as among adult cases with mild, moderate, and severe disease. IgM memory B cells were absent in moderate and severe cases whereas frequencies of B cells with a lack of surface immunoglobulin expression were significantly higher in severe cases. Interestingly, these immune subsets remained stable during recovery implying that these subsets could be associated with underlying baseline immune variation. Our results offer insights into the potential immune markers associated with severe COVID-19 and provide a foundation for future research in this area.

## Introduction

The recent SARS-CoV-2 pandemic has posed unprecedented challenges to global public health. Immunosenescence, a decline in immune function with increasing age, is a key factor contributing to severe infections, making older individuals particularly susceptible to COVID-19-related mortality^1–4^. Moreover, differences in immune responses to SARS-CoV-2 among individuals suggest that age-related changes and underlying immune variation may contribute to the heterogeneous outcomes observed in COVID-19 patients^5^. A comprehensive understanding of immune responses to SARS-CoV-2 and baseline immune cell subset differences in individuals at increased risk for COVID-19 could facilitate their early identification and better long-term strategies to protect these risk groups from severe disease.

Extensive research has been conducted to understand immune responses to SARS-CoV-2, focusing primarily on hospitalized patients and severe cases^6,7^. These studies have reported reduced frequencies of dendritic cell (DC) and natural killer (NK) subsets, as well as hyperactivation of CD4+ and CD8+ T cell compartments in severe cases^5–7^. However, the underlying inter-individual variation between mild and moderate cases, which represent the majority of cases is largely unknown^8^. In contrast, children cases often remained asymptomatic and rarely developed severe COVID-19 at the beginning of the pandemic, therefore they hold substantial significance in understanding immune responses against SARS-CoV-2^9–11^. Recent studies suggested that rapid interferon (IFN) production and lack of cytokine storm may underlie the milder disease course observed in children cases^12,13^.

In this study, we investigated the inter-individual immune variation in response to SARS-CoV-2 infection by conducting a comprehensive comparison of T cell, B cell, and innate cell subsets among infected children and adults experiencing varying disease severities (from mild to moderate, and severe). Moreover, we examined immune subset kinetics in recovering individuals to identify potential indicators of impaired immune function. These individuals had not been previously exposed to the SARS-CoV-2 virus or received vaccinations as they were included during the first wave of SARS-CoV-2 infections (March–April 2020). Our findings enhance the understanding of immune variation in the context of SARS-CoV-2 infection and highlight immune subsets potentially associated with severe disease.

## Results

### Characteristics of the study population

**Figure 1:**
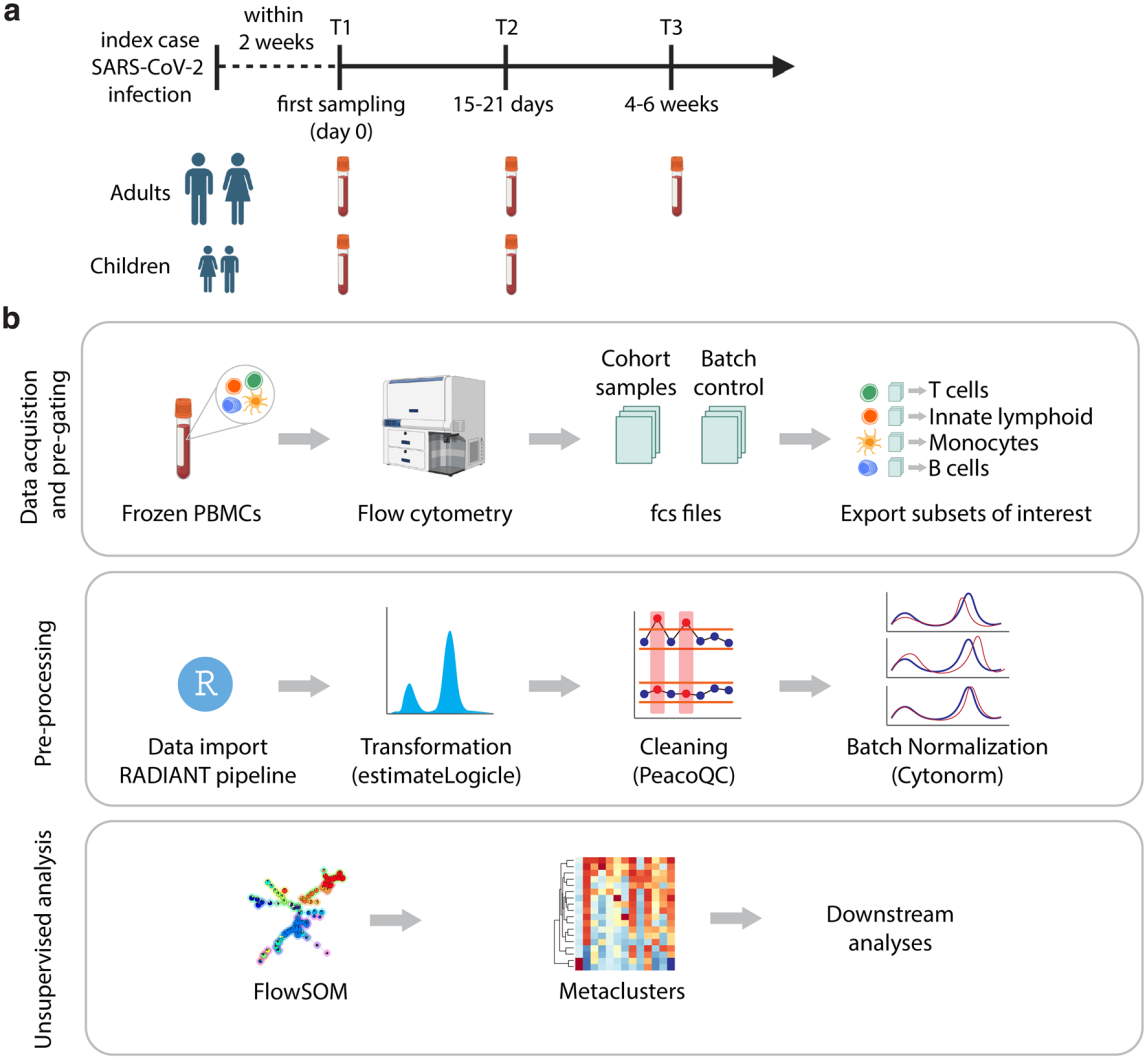
Study population and analysis pipeline. (**a**) SARS-CoV-2 infection cohort study design. (**b**) Step-by-step RADIANT pipeline description which includes data acquisition, pre-processing steps and unsupervised analysis.

We analyzed peripheral blood mononuclear cells (PBMCs) from children and adults with PCR-confirmed SARS-CoV-2 infection (Table 1). We aimed to investigate immune subset differences: (1) between 24 children and 41 adults, (2) among 57 adult samples with varying disease severity—mild, moderate, and severe, as previously described (Materials and Methods)^11^ and (3) 18 recovering adult cases who turned PCR− after T1 (Table 1). The median time point for the first sample (T1) was 11 days [interquartile range (IQR) 11 days] for children and 12 days [IQR 6 days] for adults after the onset of symptoms (Fig. 1a).

**Table 1:** Demographic and clinical features of adults and children with PCR-confirmed SARS-CoV-2 infection.

	Mild	Moderate	Severe	Total
*PCR*+ *adults and children*				
*Adults*				
n (%)	26 (63%)	9 (22%)	6 (15%)	41
Median age (IQR)	43 (36, 48)	40 (32, 45)	50 (47, 52)	
Sex—female (%)	13 (50%)	6 (67%)	2 (33%)	
Timepoint T1 (%)	23 (88%)	9 (100%)	6 (100%)	
Timepoint T2 (%)	3 (12%)	0 (0%)	0 (0%)	
*Children*				
n (%)	22 (92%)	2 (8%)	0	24
Median age (IQR)	12.0 (10.2, 14.0)	15.5 (15.2, 15.8)	NA	
Sex—female (%)	12 (55%)	1 (50%)	NA	
Timepoint T1 (%)	20 (91%)	2 (100%)	NA	
Timepoint T2 (%)	2 (9.1%)	0 (0%)	NA	
*PCR*+ *adults disease severity*				
n (%)	38 (67%)	13 (23%)	6 (10%)	57
Median age (IQR)	44 (38, 48)	45 (34, 49)	50 (47, 52)	
Sex—female (%)	20 (53%)	7 (54%)	2 (33%)	
Timepoint T1 (%)	22 (58%)	9 (69%)	6 (100%)	
Timepoint T2 (%)	16 (42%)	4 (31%)	0 (0%)	
*Recovering adults*				
n (%)	10 (56%)	6 (33%)	2 (11%)	18
Median age (IQR)	43 (36, 47)	37 (22, 42)	43 (42, 44)	
Sex—female (%)	3 (30%)	6 (100%)	1 (50%)	
Timepoint T1 (%)	10 (100%)	6 (100%)	2 (100%)	

*IQR* inter quartile range.

### Addressing technical variation and batch normalization

We implemented comprehensive flow cytometry analyses on frozen peripheral blood mononuclear cells (PBMCs) derived from COVID-19 patients to assess their immune subset composition. In these analyses, we focused on CD4+ and CD8+ T cell, B cell and innate myeloid and lymphoid subsets (Supplementary Fig. S1a–c). While technical variation in flow cytometry data is commonly managed through cytometer setup and tracking, mean fluorescence intensity (MFI)-based analysis remains susceptible to variability between batches and the quality of samples^14^. Notably, our study detected a discrepancy in laser performance across different batches (Supplementary Fig. S2a). To rectify this issue, we devised an accessible analysis pipeline named R-based Analytical tool for DIfferential ANalysis of cyTometry data (RADIANT) (Fig. 1b). This pipeline enabled us to normalize the batch effect (Supplementary Fig. S2b) and employ flowSOM, an unsupervised analysis method, to identify immune cell subset populations in our dataset, thereby ensuring accurate immune subset identification. Clusters representing immune subsets numbered for each lineage subset, CD4+ T cells (CD4), CD8+ cells (CD8), B cells (B), innate myeloid (M) and innate lymphocytes (IL) (Supplementary Fig. S3a–e).

### Infected adult and children cases show distinct immune profiles

**Figure 2:**
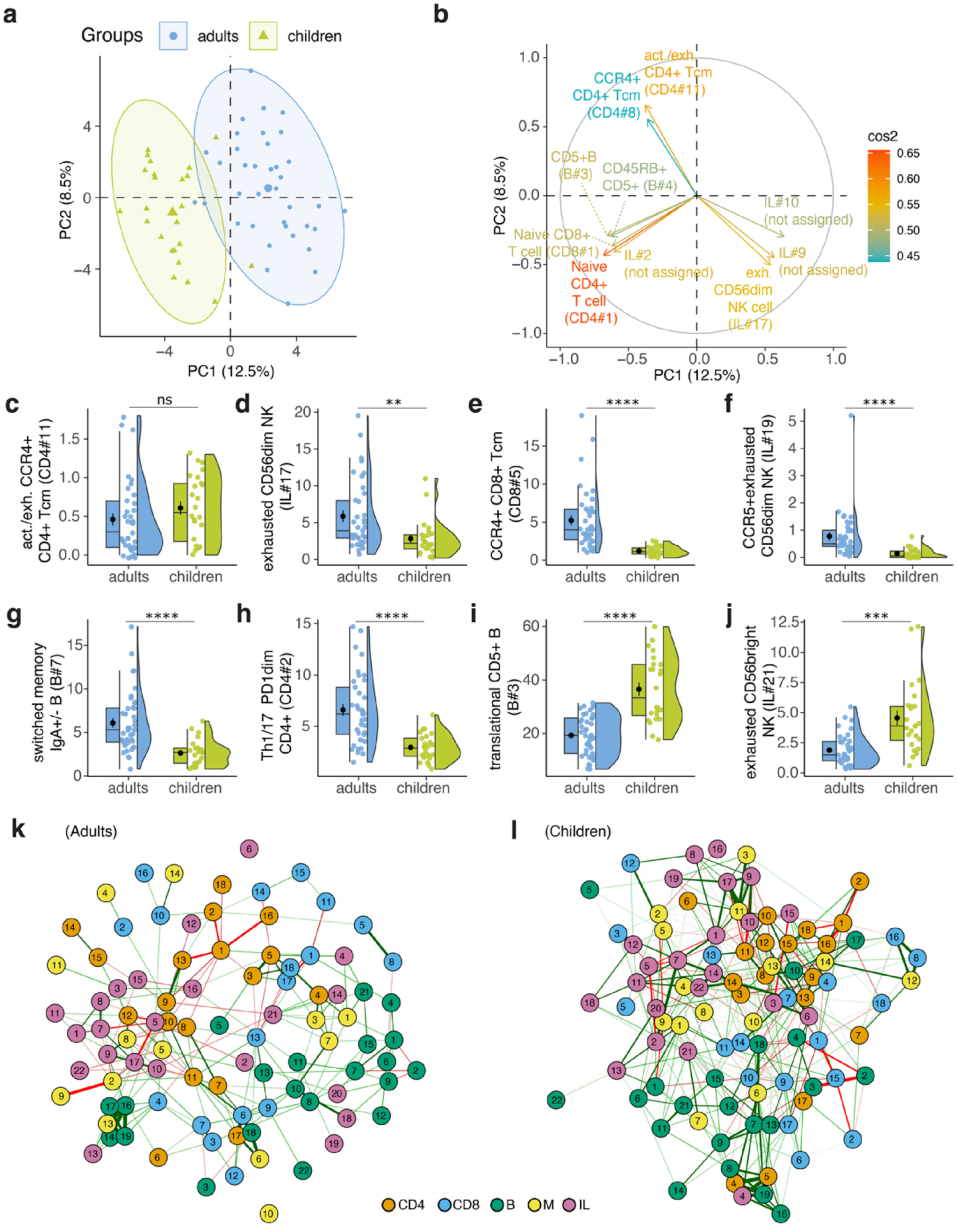
Age and SARS-CoV-2 infection-related immune subsets explain the highest variation in PCR+ adults and children. (**a**) Projection of unsupervised immune subset clusters onto the principal components (PC). (**b**) Contribution of top 10 immune subsets explaining the variance on PC1-2. (**c**–**j**) Plots depicting selected immune subsets that explain the variance om PC1-2 or are significantly different between children and adults. (**k**) Immune network correlations of adult and (**l**) children showing correlations stronger than the absolute value of 0.4 and *p* < 0.05 were shown. The numbers in nodes represent the immune subset IDs for a given cell subset type.

To elucidate overall differences in immune subsets between infected adults and children, we analyzed immune subset of individuals from the time point when they were first tested SARS-CoV-2 positive by PCR test. For both adult and children cases, the majority of samples were from T1 time point (n = 23, adults 88%, n = 20, children 91%) (Table 1). Three adults and two children samples were PCR− at T1 and seroconverted PCR+ at T2.Out of 24 children cases children, 3 cases were 5 years old or younger, and 19 cases were 10 years old or older. First, we conducted a principal component (PC) analysis using immune cell subset data acquired from unsupervised clustering (Supplementary Fig. S3a–e). A distinct segregation between infected adults and children emerged on PC1 (Fig. 2a), driven by age-related changes in the immune system, specifically CD4+ naïve T (CD45RO−CCR7+CD27+CD28+) (CD4#1) and CD8+ naïve T (CD8#1) cells (Fig. 2b, Supplementary Table S2). The observed variance between the children and adult samples could be explained by the presence of activated (CD38+CXCR3+)/exhausted (TIGIT+PD−1+) CCR4+ CD4+ T central memory (cm) cells (CD4#11) and exhausted (NKG2A+) CD56dim Natural Killer (NK) cells (IL#17), which were among the most important variables contributing the variance explained on PC1-2 (Fig. 2a,b).

We then investigated the immune subsets that exhibited statistically significant differences between infected adult and children (Supplementary Table S2). Although activated/exhausted CCR4+ CD4+ Tcm cells (CD4#11) were one of the most important variables contributing to the variation on PC1-2, this subset was not statistically significant between children and adults (Fig. 2c). On the other hand, exhausted CD56dim NK cells (IL#17), another important variable associated with the variation on PC1-2 was significantly higher in adults compared to children cases (Fig. 2d). The most pronounced difference between adults and children was in the percentage of CCR4+ CD8+ Tcm cells (CD8#5) (*p*.adj = 1.1 × 10^−8^) (Fig. 2e), which was higher in adults and indicates lung homing^15^. Percentages of CCR5+ exhausted CD56dim NK cells (IL#19), switched memory IgA+/− B cells (B#7), and Th1/17 (CXCR3+CCR6+CCR4-) PD1dim CD4+ T cells (CD4#2) were also significantly higher in adults compared to children (Fig. 2f–h). In contrast, children displayed a markedly higher percentage of translational CD5+ B cells (B#3) and exhausted CD56bright NK cells (IL#21) relative to adults (Fig. 2i,j). Percentage of naïve CD4+ (CD4#1) and naïve CD8+ (CD8#1) T cells, which show age-related decline, were also significantly higher in children^16^ (Supplementary Table S2).

To further explain our findings, we examined the correlations within the immune network for all identified immune subsets, for both adult and children cases. We used strength, betweenness and closeness centrality measures of these immune subsets in the immune network, which allowed us to gain a more comprehensive understanding of the interrelationships between diverse immune subsets and their collective responses to SARS-CoV-2 infection. Strength measures the total weight of a node's connections, betweenness centrality quantifies how often a node acts as a bridge along the shortest path between two other nodes, and closeness centrality measures how close a node is to all other nodes in the network^17^. In the adult immune network, we identified activated (CD38+CXCR3+)/exhausted (TIGIT+PD−1+) CCR4+ CD4+ T central memory (cm) cells (CD4#11) showing the highest strength and closeness (Supplementary Fig. S4a). This cell subset was not only one of the top ten most important variables that explained variance between children and adults on PCA but was also highly significantly different between these two groups. We also identified exhausted CD56dim NK cells (IL#17), which ranked within the top five for betweenness, strength, and closeness centrality measures (Fig. 2k, Supplementary Fig. S4a). These findings suggest that these immune subsets play critical roles in the immune network, potentially serving as key mediators within the network.

In children cases, CD14-CD16dim HLA-DR+ CD86+ myeloid cells (M#6), which was statistically lower in children cases (*p*.adj = 5.7 × 10^−3^), and CD11cdimCD27+ CD24-CD38hi B cells (B#18) both ranked within the top two scores for betweenness and closeness centrality measures, indicating their importance in the immune network of children (Fig. 2l, Supplementary Fig. S4b). Taken together, the key insights from these analyses between adults and children underscore the absence of severe infection in children cases, as evidenced by the lack of CCR4+ CD8+ T cells, in conjunction with variations in CD56 expression in the exhausted NK cell phenotype.

### Immune subset differences in disease severity groups

**Figure 3:**
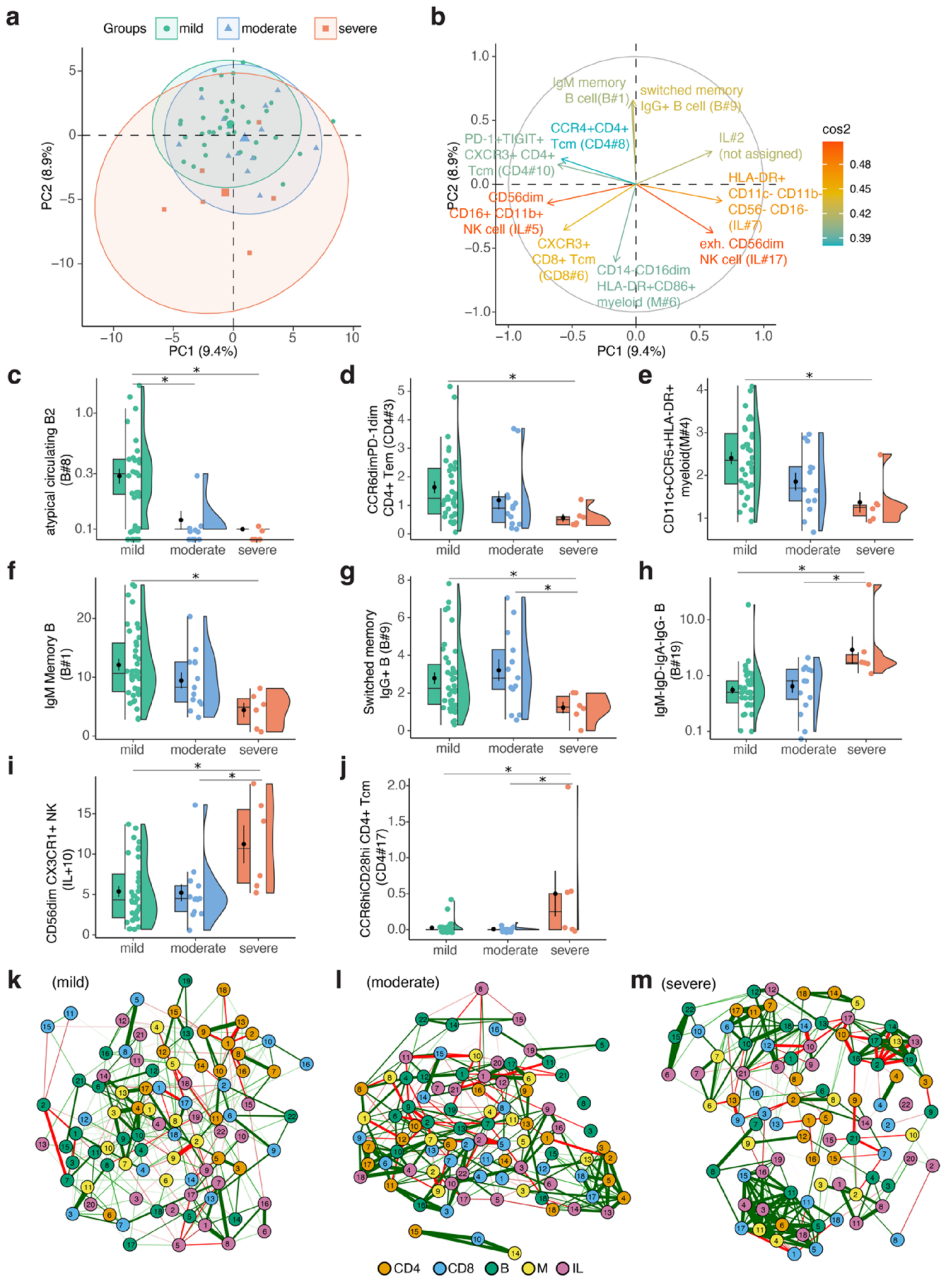
Increased immune variation in higher disease severity driven by exhausted immune subset phenotypes. (**a**) Projection of unsupervised immune subset clusters onto the principal components (PC). (**b**) Contribution of top 10 immune subsets explaining the variance on PC1-2. (**c**–**j**) Plots depicting selected immune subsets that are significantly different between severity groups**.** Benjamini–Hochberg corrected *p* values are reported. (**k**) Immune network correlations of mild, (**l**) moderate and (**m**) severe adult cases depicting correlations stronger than absolute value of 0.4 and *p* < 0.05 were shown. The numbers in nodes represent the immune subset IDs for given cell subset type.

Next, we further investigated the association of immune subsets with COVID-19 disease severity. We analyzed 57 PCR+ adult samples from T1 and T2 time points with varying severity (Table 1). Among these, 16 adults had samples both from T1 and T2 time points. Between T1 and T2, three adults changed from severe severity to moderate, and two adults changed from moderate to mild severity. The rest of the cases remained in the same category levels (mild = 10, moderate = 1). Then, we performed a PC analysis to compare immune subset variables among adults with disease severity varying from mild to moderate and severe. Although the variance in PC1-2 did not lead to a distinct separation between disease severity groups, we observed a greater dispersion in PC1-2, with increased disease severity, (Fig. 3a). This indicates that higher disease severity is associated with higher immune variation. Immune subsets that contributed to the variance the most were not statistically significant between disease severity groups, which could be due to very limited variation captured by PCA (Fig. 3b).

Next, we zoomed in on the immune subsets that were statistically significant between disease severity groups (Table 2). In mild cases, we observed a significantly higher percentage of atypical (CD11c+) circulating B2 (IgD+CD27+) B cells (B#8) compared to severe cases (Fig. 3c). CD11c expression is a canonical marker of Atypical B Cells (ABC)^18^ whereas previously IgD+ CD27+ cells were reported as high-affinity IgM-producing subset^19^. Additionally, percentages of CCR6dimPD-1dim CD4+ T effector memory (em) cells (CD4#3), CD11c+ CCR5+ HLA-DR+ myeloid cells (M#4), and IgM memory B cells (IgM+IgD+CD27+) (B#1) were significantly higher in mild cases than in severe cases but not moderate cases (Fig. 3d–f). Among these subsets, IgM+ memory B cells are reported as a heterogeneous subset that showed an age-related decline and T cell independent function potential^20,21^.

**Table 2:** Significantly different immune subsets between disease severity groups.

Immune subset	Variable	Group1	Group2	*p*.adj	*p*.adj.signif
IgM-IgD-IgA-IgG- B cell	B#19	Mild	Severe	0.001	**
IgM memory B cell	B#1	Mild	Severe	0.004	**
CD11cdimCD27+ CD24-CD38hi B cell	B#18	Mild	Severe	0.005	**
IgM-IgD-IgA-IgG- B cell	B#19	Moderate	Severe	0.009	**
CCR6hiCD28hi CD4+ Tcm	CD4#17	Mild	Severe	0.012	*
CCR6hiCD28hi CD4+ Tcm	CD4#17	Moderate	Severe	0.012	*
Atypical circulating B2 B cell	B#8	Mild	Moderate	0.012	*
CD11c+CCR5+HLA-DR+ myeloid cell	M#4	Mild	Severe	0.013	*
CD11cdimCD27+CD24−CD38hi B cell	B#18	Moderate	Severe	0.013	*
atypical circulating B2 B cell	B#8	Mild	Severe	0.014	*
CD56dim CX3CR1+ NK cell	IL#10	Mild	Severe	0.025	*
CD14-CD16dim HLA-DR+CD86+ myeloid	M#6	Mild	Severe	0.026	*
CD56dim CX3CR1+ NK cell	IL#10	Moderate	Severe	0.03	*
CCR6dimPD-1dim CD4+ Tem	CD4#3	Mild	Severe	0.03	*
Not assigned	CD4#12	Mild	Moderate	0.044	*
Not assigned	IL#20	Moderate	Severe	0.046	*
Switched memory IgG+ B cell	B#9	Mild	Severe	0.047	*
Switched memory IgG+ B cell	B#9	Moderate	Severe	0.047	*
Translational CD5+ B cell	B#3	Moderate	Severe	0.047	*

*p.adj ≤ 0.05, **p.adj < 0.01.

Switched memory IgG+ B cells (B#9) showed a significantly lower percentage in severe cases compared to moderate cases (Fig. 3g). In contrast, severe cases demonstrated a significant elevation in percentage of unusual B cells characterized by a lack of surface immunoglobulins (IgD−IgM−IgG−IgA−) (Igs−) CD38+CXCR5− expression profile (B#19), CD56dim CX3CR1+ NK cells (IL#10), and CCR6hiCD28hi CD4+ Tcm cells (CD4#17) compared to both mild and moderate cases (Fig. 3h–j). CX3CR1 expression marks potent cytotoxicity in NK cells and was reported to be higher in severe COVID-19 cases^5,22^.

We constructed immune network correlations for adults with varying disease severity to elucidate differences in immune network organization and to identify key immune subsets associated with each severity level (Fig. 3k–m). However, the immune subsets that scored highest for betweenness, strength, and closeness centrality measures were not included among the subsets showing significant differences across severity levels, nor were they among the top 10 immune subsets explaining the variance in PCA (Supplementary Fig. S5a–c). Interestingly, as disease severity increased, we observed a higher density of nodes with stronger correlations, reflected by an increase in both strength and closeness centrality measures. Such a network may represent a concerted effort by the immune system to counteract the infection, thus leading to a heightened intensity of immune interaction in more severe cases.

### Immune subset kinetics in recovering individuals

**Figure 4:**
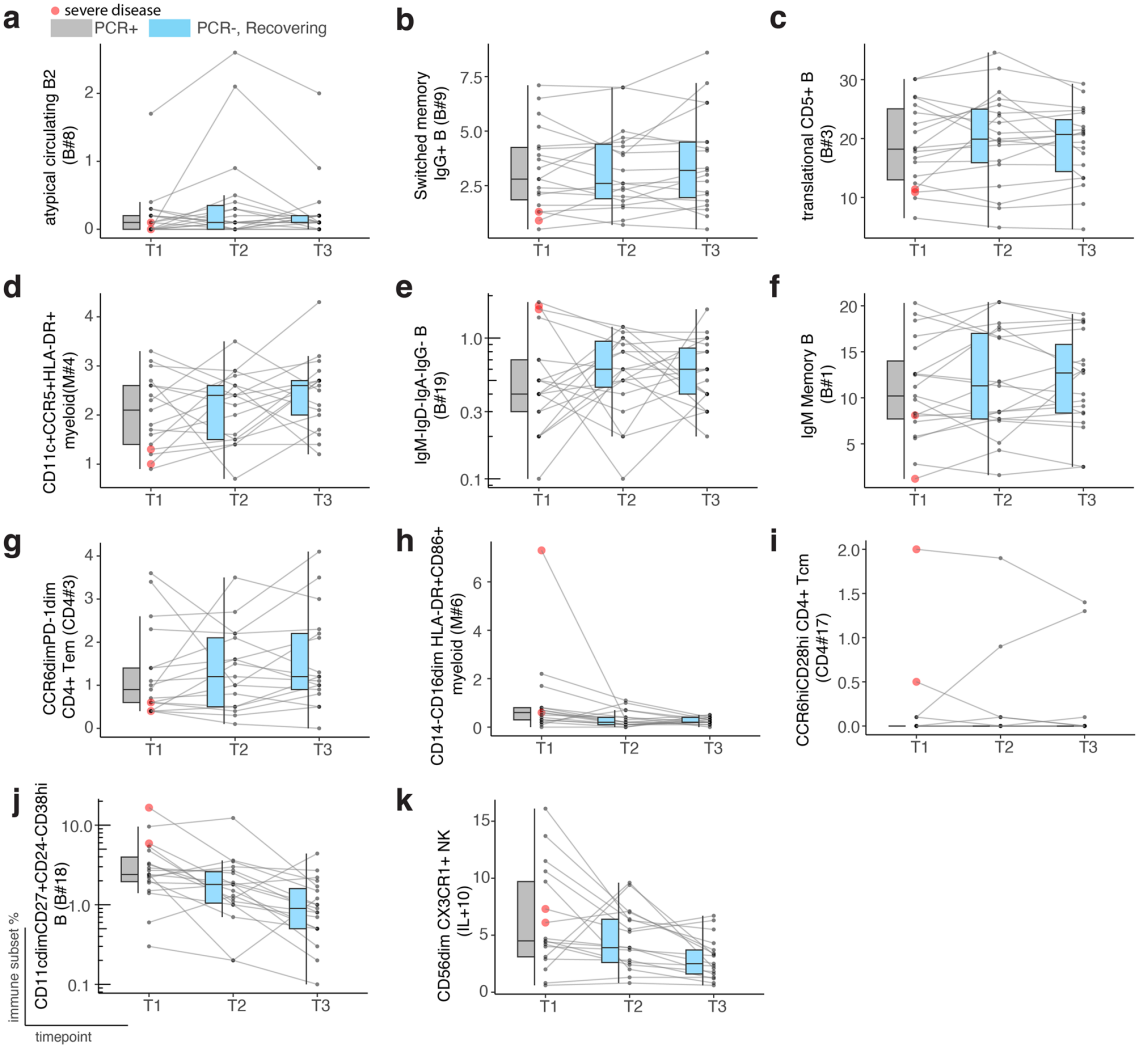
Immune subsets linked to disease severity exhibit consistent kinetics during recovery, potentially reflecting underlying immune variation. (**a**–**k**) Immune subsets correlated with disease severity are depicted across three timepoints. T1 (grey) represents PCR+ samples, while T2–T3 display (blue) PCR- recovering samples for each individual. Red circle represents severe adult cases at T1.

To determine whether immune subsets with significant differences between disease severity groups were associated with infection or were a result of underlying baseline immune signatures, we tested the kinetics of these immune subsets (Table 2) in 18 recovering adult cases (defined as PCR+ at T1 and PCR- for T2 and T3, Table 1). Immune subsets that were stable during the recovery phase (repeated measures ANOVA, *p* > 0.05), were percentage of atypical circulating B2 cells (B#8) and switched memory IgG+ B cells (B#9) (Fig. 4a,b). Although the percentage of translational CD5+ B cells (B#3), CD11c+CCR5+HLA-DR+myeloid cells (M#4), Igs- B cells (B#19), IgM memory B cells (B#1), and CCR6dimPD-1di CD4+ Tem cells (CD4#3) increased, and CD14-CD16dim HLA-DR+CD86+ myeloid cells (M#6) decreased in their median during recovery, these differences remained statistically stable (*p* > 0.05) (Fig. 4c–h). This indicates that these subsets could be attributed to an underlying baseline signature rather than infection-related perturbation.

CCR6hiCD28hi CD4+ Tcm cells (CD4#17) were only abundant in severe cases and for those individuals the subset kinetics during recovery was not consistent (Fig. 4i). However, the decline in the median during recovery for CD11cdimCD27+CD24−CD38hi B cells (B#18) (*p* = 7.1 × 10^−3^) and CD56dim CX3CR1+ NK cells (IL#10) (*p* = 3.0 × 10^−3^) and were statistically significant (Fig. 4j,k), implying that these subsets are infection-related.

## Discussion

In this study, we provided a comprehensive analysis of immune cell subset variation and key immune subset network variables between adult and children SARS-CoV-2 cases, as well as adults exhibiting varying degrees of disease severity. Additionally, we examined the immune subset kinetics of individuals recovering from COVID-19 to investigate potential indicators of immune dysfunction. Our findings improve our understanding of immune variation in SARS-CoV-2 infected cases and highlight immune subsets playing a key role in disease severity.

While age-related immune cell subset differences between adult and children cases were the most prominent, our findings also shed light on distinct immune responses to SARS-CoV-2 infection severity within the adult group. In adult cases, an increased percentage of lung-homing CCR4+CD8+ T cells and exhausted cytotoxic CD56dim NK cells may indicate a more potent cytotoxic response to infection^23^. However, the exhaustion of cytotoxic NK cells could also signify a reduced ability to effectively control viral replication^23–25^. Conversely, children cases exhibited a significantly lower proportion of lung-homing CD8+ T cells, suggesting weaker adaptive immune responses^13^. Intriguingly, children's exhausted NK cell profile primarily consisted of the CD56bright subset, which is capable of producing high levels of interferon (IFN) and tumor necrosis factor^26^. Given that IFN has been demonstrated to inhibit neutrophil infiltration into the lungs and prevent neutrophil-induced disease pathology, exhausted CD56bright NK cells may suggest robust IFN production early during infection^12,25^. We also observed an increased percentage of CD5+ translational B cells in children cases compared to adult cases. The role of CD5 expression on B cells remains unclear. However, in mice and ferrets, CD5+ B−1 cells have been shown to respond to influenza infection in an innate-like manner, suggesting that these cells could potentially play a role in SARS-CoV-2 infection as well^27^. Immune network correlation analysis further emphasized the importance of immune subsets based on their centrality scores. In adults, exhausted CD4+ and CD56dim NK cells exhibited the highest centrality scores, which were absent in children cases. This means that these cells are central and crucial components of the immune response in adults, whereas these cells were not prominently central in children cases.

Although COVID-19 severity has been extensively studied in severely hospitalized patients, the majority of patients exhibit mild to moderate symptoms^8^. In accordance with recent literature, we observed the most significant differences in severe cases to be increased frequencies of immune subsets with lung-homing markers, such as CX3CR1 in NK cells, CCR4 in T cells, but also exhausted CD8+ T cells^5,28^. Intriguingly, we detected a small population of B cells exhibiting atypical B cell phenotype exclusively in mild cases, with no presence in moderate or severe cases. CD11c+ B cells, primarily described as a memory B cell population, were initially reported in autoimmune diseases^29^. It is improbable that these cells are memory B cells against SARS-CoV-2, as the patients in our study had not previously been exposed to SARS-CoV-2. Nonetheless, other researchers have reported the existence of pre-existing cross-reactive B cells in some individuals^30^. Given that we observed these cells solely in mild cases, which may have inhibited progression to moderate or severe disease due to a robust memory B cell response, we propose that atypical circulating B2 cells could represent such pre-existing cross-reactive B cell subsets.

Increasing evidence highlights that immune function arises from complex interactions within the immune network, and the baseline state of this network is associated with its functional potential^31,32^. Consequently, individuals at risk of developing severe disease could be identified by examining variations in their baseline immune network^16,33^. One limitation of our study is the lack of pre-infection baseline samples, which would have allowed us to investigate baseline immune variation in our cohort. However, it has been demonstrated that the human immune system rapidly reverts to its baseline state after infection or vaccination, and the post-baseline composition is similar to that of the initial baseline^34^. For SARS-CoV-2 infection, other researchers have demonstrated that immune perturbations especially in severe cases, may persist for up to 60 days following symptom onset^35^. Although some studies have reported even more long-term immune perturbations in patients following COVID-19 recovery, these alterations are generally infection-related signatures, such as CD38+HLA-DR+ T cells and CXCR3 expression^35,36^. While we cannot rule out the presence of such persistent perturbations in our study, the risk is likely low since our cohort does not include any individuals with highly severe disease that require intensive care unit admissions or ventilation machines.

Therefore, in our study to investigate immune subsets that may potentially be linked to underlying baseline immune variation and disease severity, we focused on immune subsets that were significantly associated with disease severity but remained stable during the recovery period of approximately 2 months and did not have clear viral infection-related phenotypes. Intriguingly IgM memory B cells, also previously reported as a controversial B cell population were suggested to exhibit age-related decline and play an important role in protection against T cell independent antigens such as pneumococcal antigens^20,21^. Furthermore, others showed that pneumococcal abundance was associated with an increased risk of SARS-CoV-2 infection and delayed clearance of the virus^37^. We observed that IgM memory cells were significantly lower in severe cases and their kinetics were stable during recovery. Therefore, a lack of IgM memory cells could be a baseline signature associated with an increased risk of severe disease. On the other hand, percentage of exhausted (PD−1+TIGIT+) CD8+ Tem cells was significantly higher in severe compared to moderate cases and remained stable during recovery. These cells were previously reported as immune checkpoint blockade targets with promising results in cancer patients^38,39^. TIGIT expression alone is also reported as a sign of immunosenescence in CD8+ T cell compartment and shown to increase with chronological age previously^40^. Therefore, PD-1 and TIGIT co-expression could be associated with CD8+ T cell dysfunction and a potential risk factor for severe disease. Another subset that showed stable kinetics, and was significantly higher in severe cases was Igs- B cells. Lack of surface immunoglobulins on B cells was previously reported in B cell malignancies, however, to our knowledge participants of this study did not have such conditions^41^.

Our research also faces several additional limitations that must be acknowledged. Primarily, the limited sample size, particularly in the context of severe cases among adults, constrains the generalizability of our results. This caveat is crucial as the findings might not fully represent the broader population. Additionally, the timing of the study, conducted during the initial phase of the pandemic, is a significant factor. The samples from individuals in our cohort were collected roughly one week following the onset of symptoms. This timing is likely a key factor in our observation of a more prominent presence of adaptive immune cell subset signatures in our analysis, as opposed to innate immune responses. It's plausible that the innate immune signatures may diminish or become less discernible in some individuals during the later stages of the infection. Therefore, while our study offers valuable insights, these constraints should be carefully considered when interpreting the findings.

In summary, we identified key differences in immune subsets between adult and children SARS-CoV-2 infected cases, and between mild, moderate and severe adult cases offering insights into the underlying immune variation potentially associated with disease severity. While the individual subsets reported in this study cannot fully explain the complexity of the immune network, immune function, and disease severity, they do serve as intriguing signatures that may contribute to future research and improved identification of individuals at risk for severe disease and immune dysfunction.

## Materials and methods

### Cohort description

This study was adapted from World Health Organization First Few Hundred protocol to study SARS-CoV-2 outbreak in the Netherlands as described previously^10,11^. In short, individuals who tested PCR positive for SARS-CoV-2 between March 2020–April 2020 and their household members with at least one child that goes to primary school were recruited to the study. Nasopharyngeal/oropharyngeal swabs for PCR analysis and blood samples for immune subset analysis were collected longitudinally. None of the participants were admitted to the intensive care unit, nor previously vaccinated against SARS-CoV-2. Peripheral blood mononuclear cells (PBMCs) were isolated from heparinized blood via density gradient centrifugation over Ficoll-Hypaque (Pharmacia Biotech) and cryopreserved at − 135 Celcius. A classification of COVID-19 as mild, moderate, or severe was established based on either self-reported symptoms or the necessity of hospital admission as reported elsewhere in detail^11^.

### COVID-19 disease severity

Participants were classified as symptomatic if they experienced any of the following at any time point: respiratory issues (such as sore throat, cough, difficulty breathing, or runny nose), fever, chills, headache, loss of smell or taste, muscle or joint pain, diarrhea, nausea, vomiting, appetite loss, or fatigue. The study categorized COVID-19 into mild, moderate, and severe categories based on the participant's self-reported symptoms or if they were hospitalized. Mild cases of COVID-19 were defined as those with confirmed laboratory results and any clinical symptoms. Moderate cases displayed clinical signs of pneumonia, including shortness of breath. Severe cases involved shortness of breath and either consulting a healthcare professional, such as visiting an emergency room, or being hospitalized due to COVID-19. Out of 6 severe cases, 3 were admitted to hospital but did not require intensive care units.

### Immune cell subset phenotyping

Cryopreserved PBMCs were used for in-depth immune subset profiling stained using the following anti-human fluorochrome-conjugated antibodies: CCR2, CCR4, CCR5, CCR6, CCR7, CD11b, CD11c, CD14, CD19, CD24, CD27, CD28, CD3, CD38, CD4, CD45RB, CD45RO, CD5, CD56, CD57, CD8, CD80, CD86, CD95, CX3CR1, CXCR3, CXCR5, HLA-DR, IgA, IgD, IgG, IgM, NKG2A, NKG2C, PD-1, TIGIT, Viability (Supplementary Table S1). Samples were acquired on a 5-laser FACSymphony™ A3 Cell Analyzer (BD Biosciences).

### Flow cytometry data analysis

Singlets and live cells were gated using FlowJo (V10.7.1 Tree Star). CD4+ T cell, CD8+ T cell, B cell, Innate lymphocyte, and Myeloid cell populations were exported. For each batch, technical controls from the same donor were acquired to be used for batch normalization. Downstream analyses were performed in R studio using R-based Analytical tool for DIfferential ANalysis of cyTometry data (RADIANT) pipeline, a user-friendly publicly available analysis pipeline developed in-house. For data transformation flowWorkspace package, for data cleaning and quality control PeacoQC, for batch normalization cytonorm and for unsupervised analysis flowSOM libraries were used. Metacluster sizes were determined by investigating cluster separation in UMAPs and marker expression profiles of metaclusters in heatmaps. Abundances of flowSOM metaclusters for each cell population were exported for the downstream analysis.

### Statistical analysis

All analyses were performed using R (version 4.2.2) and RStudio (version 2022.12.0.353). The packages FactoMineR (version 2.7) was utilized for principal component analysis, rstatix (version 0.7.2) for other statistical tests, and qgraph (version 1.9.4) for network analysis. Four adults who had missing flow cytometry data were therefore excluded from principal component analyses. Differences between infected adult and children samples were assessed using the Mann–Whitney U test, while distinctions among disease severity groups in adults were evaluated with the Kruskal–Wallis test, followed by Dunn's post hoc test. The Benjamini–Hochberg (BH) procedure was applied to correct for multiple testing, and the adjusted *p* values are denoted as “*p*.adj” in the reported results. Repeated measures ANOVA was used to evaluate differences in immune subset kinetics during recovery. Boxplots display the median and interquartile range (IQR) (25–75%), with whiskers representing the upper- and lower-quartile ±1.5 × IQR. Statistical significance levels in figures are designated as follows: ns > 0.05 *p.adj ≤ 0.05, **p.adj < 0.01, ***p.adj < 0.001, ****p.adj < 0.0001.

### Ethics approval and consent to participate

The University Medical Center Utrecht Medical-Ethical Review Committee approved the generic and adapted study protocols (NL13529.041.06). All procedures were performed in compliance with Good Clinical Practice and in accordance with the principles of the Declaration of Helsinki. All participants aged > 12 years provided written informed consent prior to study onset; parents or guardians of participating children aged < 16 years gave written informed consent for participation; for children aged 12–16 years both parents and children gave consent.

### Supplementary Information

Supplementary Information 1.

Supplementary Information 2.

Supplementary Information 3.

## Data Availability

Datasets are available from Dr. Jelle de Wit (corresponding author) upon request with consideration of the participants’ privacy rights.

## Code availability

All the custom codes used for the analysis reported are available on GitHub: https://github.com/alpercevirgel/FFX-SARS-CoV-2. Flow cytometry data analysis pipeline RADIANT is available on https://github.com/alpercevirgel/RADIANT.
