# Supplementary material for: Delineating immune variation between adult and children COVID-19 cases and associations with disease severity
Source: Sci Rep. 2024 Mar 1;14:5090. doi: 10.1038/s41598-024-55148-9 (PMC10907598; doi:10.1038/s41598-024-55148-9)
Supplement: Supplementary file 1 — Supplementary Information 1. [file 41598_2024_55148_MOESM1_ESM.docx]

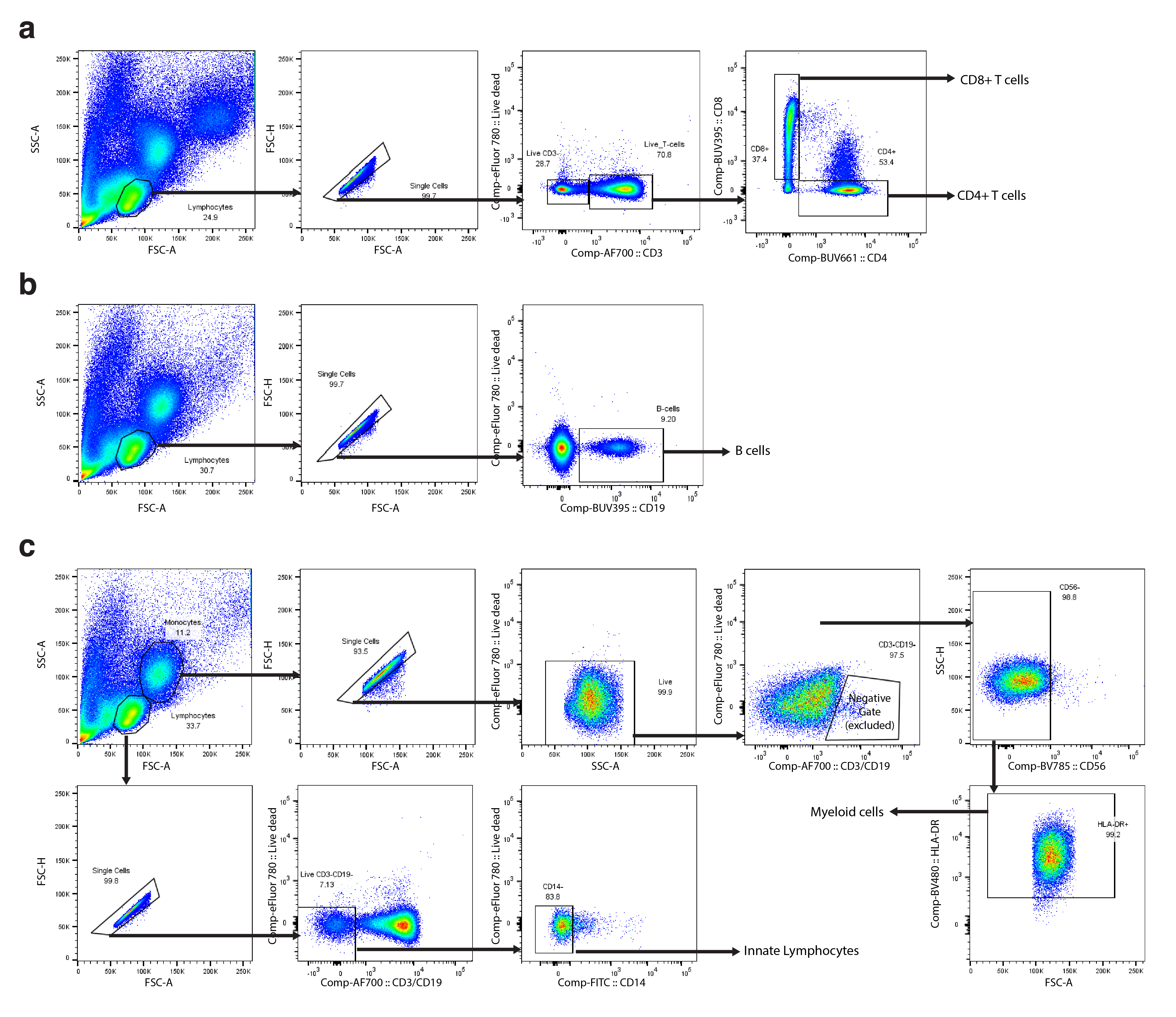


**Supplementary Figure 1:** Gating strategy of the immune subset that are imported into RADIANT pipeline for unsupervised analysis. **(a)** CD4+ and CD8+ T cells, **(b)** B cells, **(c)** Myeloid and innate lymphocytes.


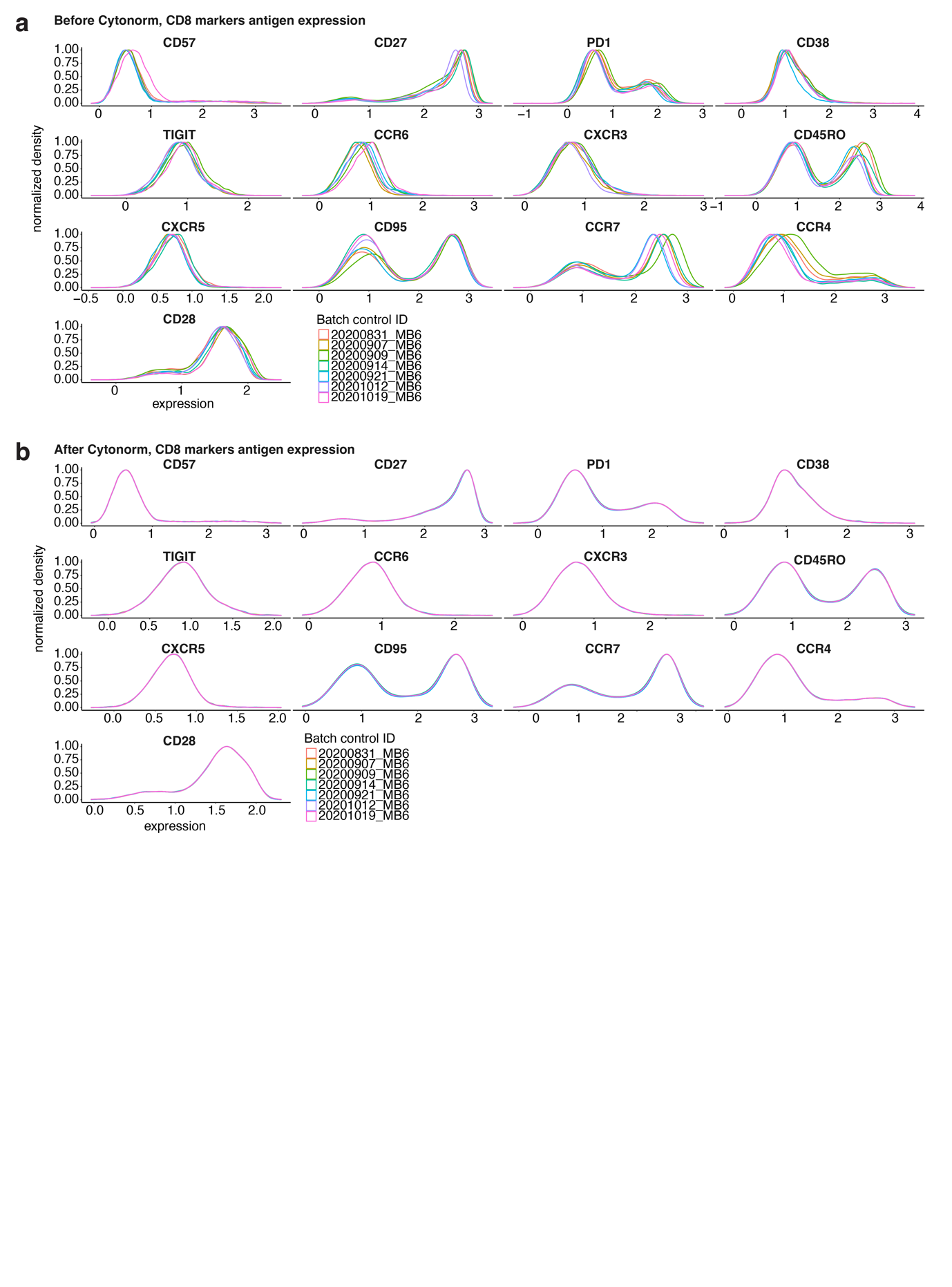


**Supplementary Figure 2:** CD8 subset marker expressions were given as an example for **(a)** before and **(b)** after batch normalization by cytonorm. Different colors indicate the batch controls.

**
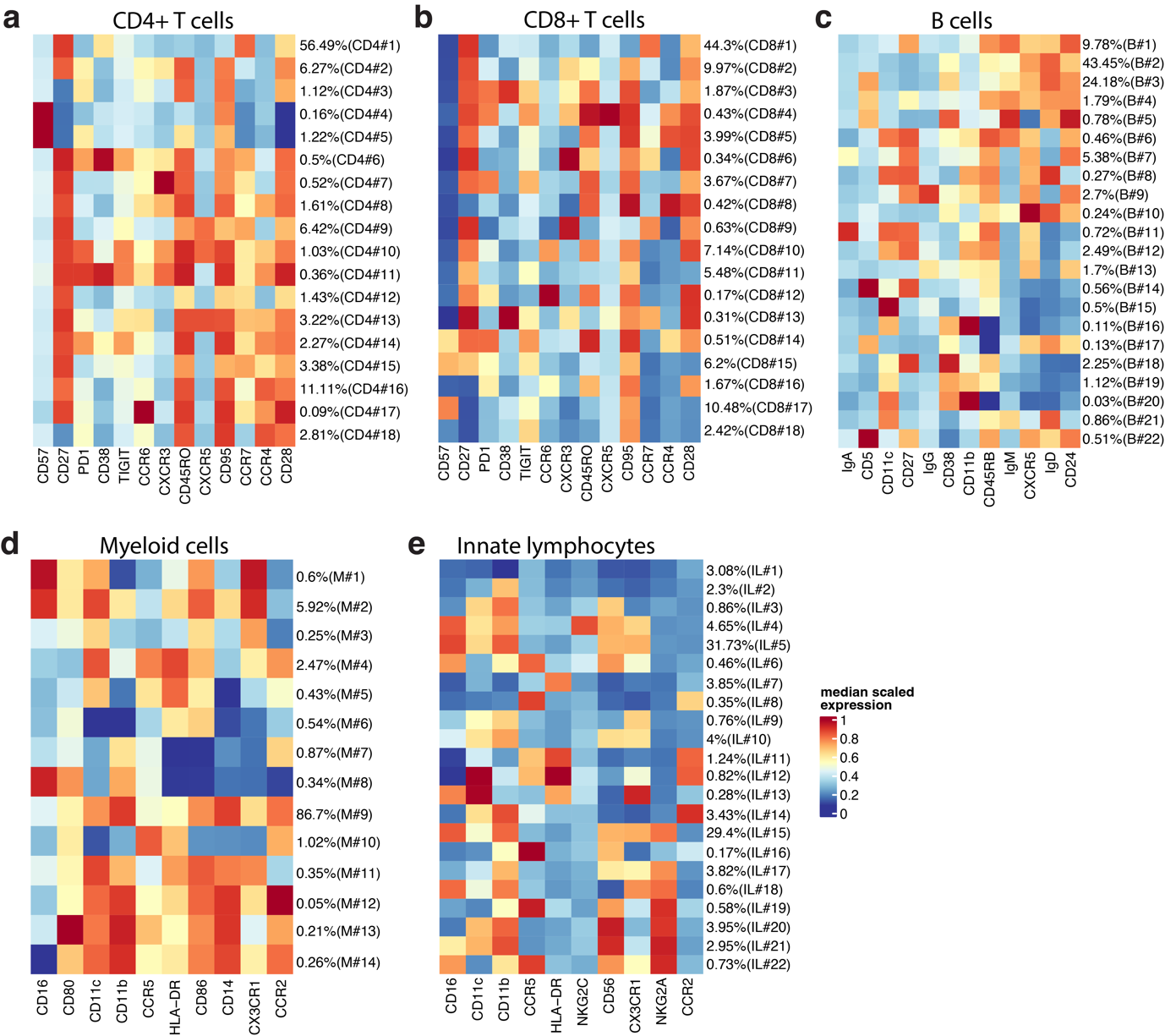
Supplementary Figure 3:** **Unsupervised clustering reveals adaptive and innate immune cell subset populations. (a)** CD4+ T cell, **(b)** CD8+ T cell, **(c)** B cell, **(d)** Myeloid cells and **(e)** Innate lymphocytes obtained from flowSOM clustering represented on heatmaps. Row represents immune cell subset populations with their percentages and columns describe surface antigens for each subset. Colors indicate median scaled expression for antigens.

**
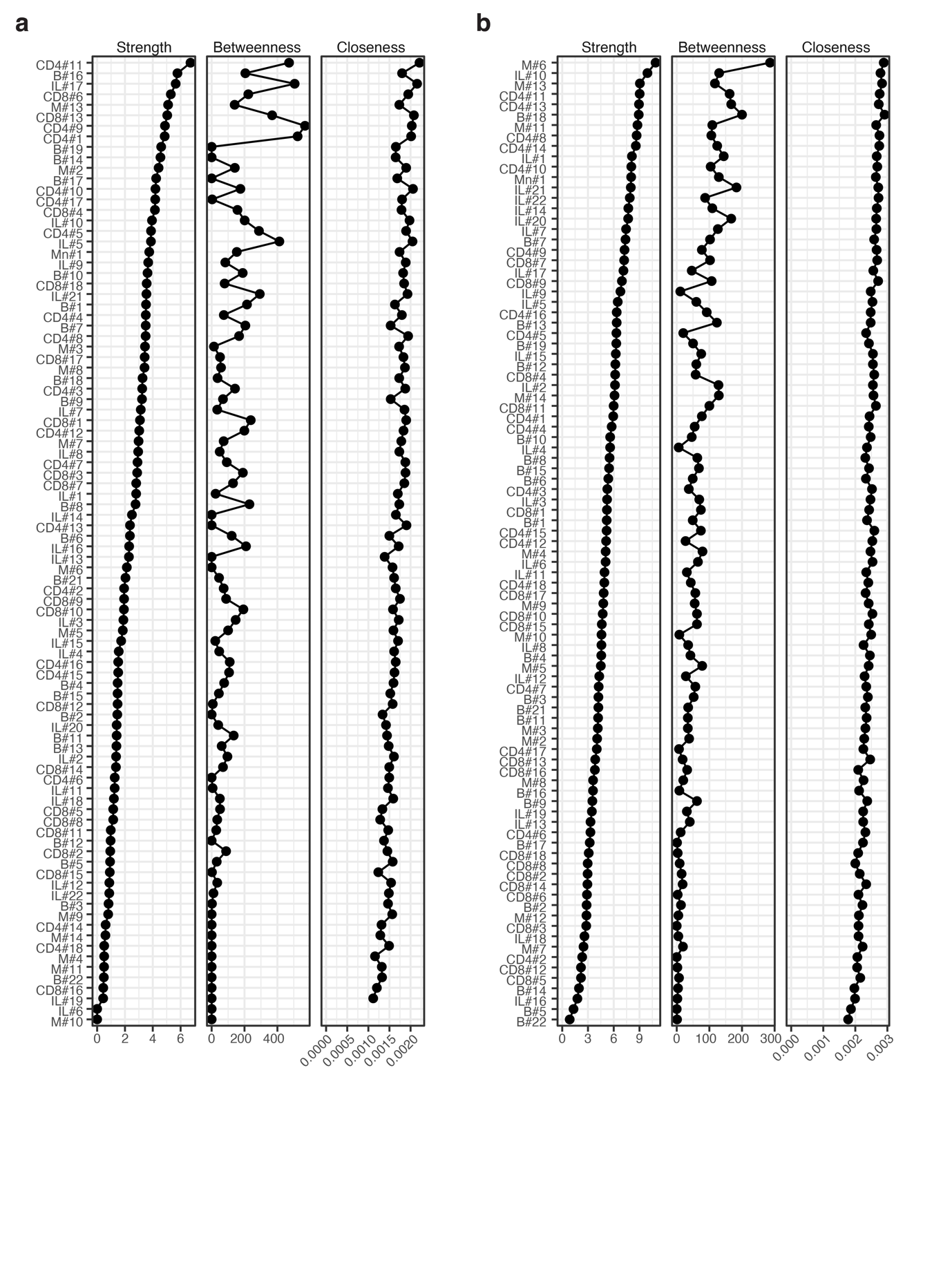
Supplementary Figure 4:** Strength, betweenness and closeness centrality measures for **(a)** adult and **(b)** children immune networks


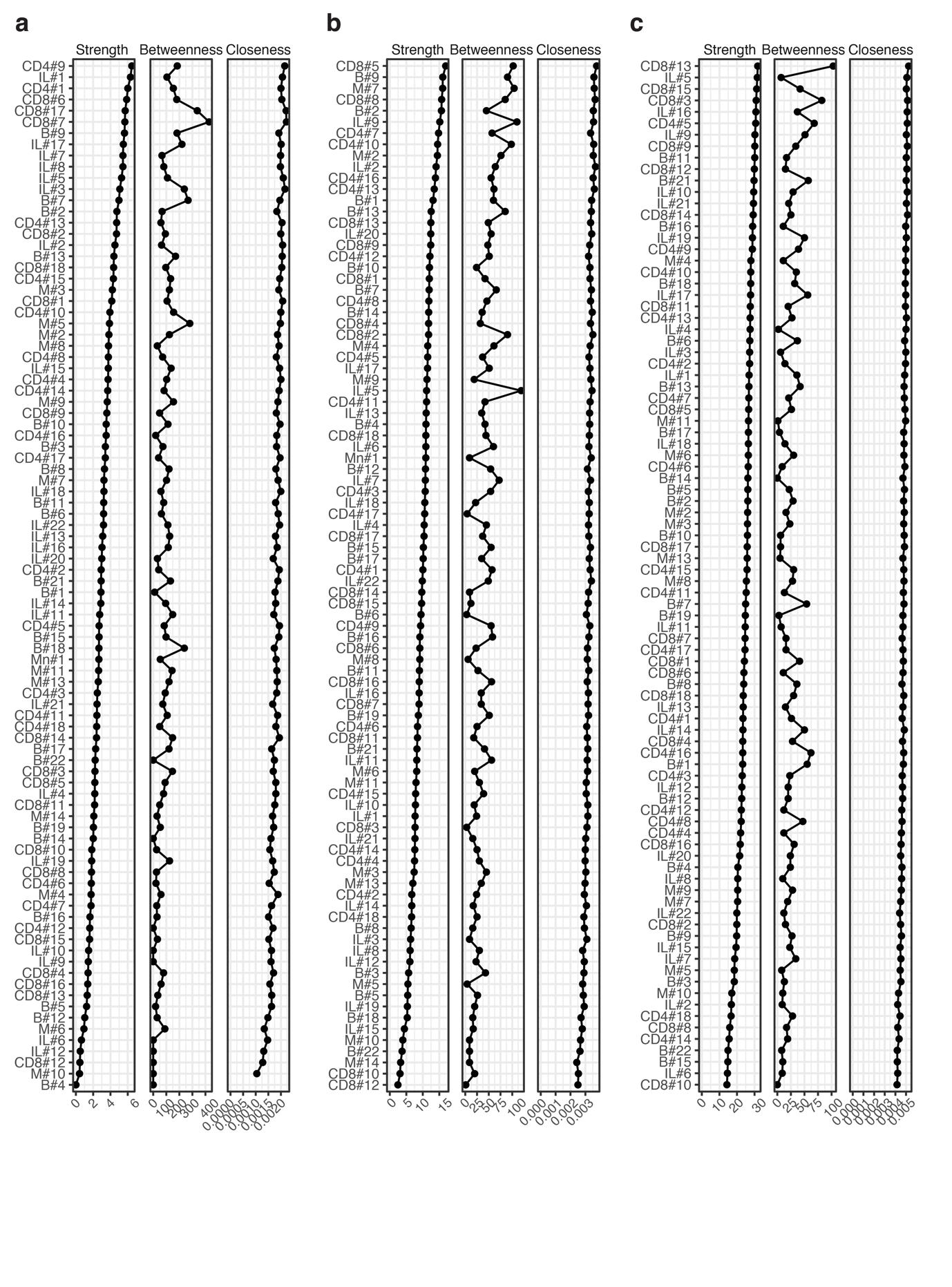


**Supplementary Figure 5:** Strength, betweenness and closeness centrality measures of immune networks for **(a)** mild **(b)** moderate and **(c)** severe adult cases.

**Supplementary Table S1:** Anti-human fluorochrome-conjugated antibody clone, supplier and panel information.

**Supplementary Table S2:** Significantly different immune subsets between infected adults and children
